# Framework for uncertainty quantification of wave–structure interaction in a flume

**DOI:** 10.1007/s40571-025-00967-4

**Published:** 2025-05-22

**Authors:** Xiaoyuan Luo, Vijay Nandurdikar, Sang-ri Yi, Alistair Revell, Georgios Fourtakas, Ajay B. Harish

**Affiliations:** 1 School of Engineering, University of Manchester, Manchester, UK; 2 NHERI SimCenter, University of California, Berkeley, CA USA

**Keywords:** Wave attenuation, Smoothed-particle hydrodynamics, Latin hypercube method, Wave flume, Structural response, Uncertainty quantification

## Abstract

In this paper, we propose a numerical procedure for the quantification of uncertainties in wave–structure interaction. We utilize the smoothed particle hydrodynamics (SPH) scheme for modeling the wave mechanics, coupled one-way with a finite element method (FEM) for the structural response. Physical wave flumes are extensively used in the study of hydrodynamics especially in wave–structure interaction (WSI) and prediction of forces to near-shore structures in disaster mitigation and offshore structures in the oil and gas, and more recently renewable energy sector. Over the years, numerical wave flumes have been developed extensively to enable the modeling of complex wave–structure interaction. However, most of these studies are deterministic and limited to using either simple flexible beams or rigid monolithic structures to model the structural part in the WSI. Additionally, uncertainties are commonly observed in both wave and structural parameters and need to be accounted for. This work presents a numerical framework to enable uncertainty quantification for wave–structure interaction problems in terms of the forces experienced by the structure. A one-way coupling between SPH with the FEM and uncertainty quantification is proposed. We employ the so-called Tokyo wave flume geometry, which has potential for future surrogate modeling in WSI. The developed model is validated using numerical and experimental results from the literature and is used to demonstrate the prediction of probabilistic responses of structures under breaking and non-breaking wave scenarios.

## Introduction

Water-borne hazards, particularly tsunamis, storm surges, and inland and coastal flooding events, are known for their devastating effects. The 2004 Indian Ocean tsunami caused waves up to 30 m high, resulting in the deaths of over 250,000 people. Similar large-scale destruction was observed with damage to ports and infrastructure inflicted by the 2010 Chile and 2011 Tohoku tsunamis, oil rigs and other infrastructure during the 2005 Hurricane Katrina storm surge, and large-scale flooding events attributed to adverse weather effects in parts of continental Europe and India during the summer of 2023. Many of these events, once considered to occur only once a decade, are happening more frequently. This increase is expected to continue due to climate change and increased human activity in coastal areas. Therefore, understanding the impact of these extreme phenomena on the urban built environment remains paramount to improving structural resilience. Furthermore, there is a clear drive for clean energy in offshore renewable structures such as wind turbines and hybrid wind and current offshore platforms.

The value of experimental wave flume campaigns for coastal engineers in systematically studying wave–structure interaction (WSI) has been well demonstrated. In recent years, a wide range of coastal engineering problems have been investigated using wave flume experiments, including beach erosion and accretion, the design of breakwaters and seawalls, wave impact on offshore structures, and sediment transport by waves [9, 41, 51]. However, the construction and operation of wave flumes can be expensive and time-consuming, particularly when investigating different scenarios. Additionally, careful design and scaling of flume experiments are necessary to ensure their real-world relevance. To reduce the cost of experimental campaigns, especially during the design phase, numerical models can be employed to assist with the design of wave flume experiments. This approach can also serve to validate the accuracy of the numerical models themselves.

In an effort to replicate the experiments by computer simulations, digital wave flumes were developed using numerical methods. Common methods and tools that have been adopted include potential flow solvers (e.g., OceanWave3D [15]), shallow water solvers (e.g., GeoClaw [6, 24], AdCirc [29]), finite volume (FVM) solvers (e.g., OpenFOAM [7, 19, 21]), and mesh-less solvers (e.g., smoothed particle hydrodynamics (SPH) [1–3, 46]). A comprehensive review of developments in WSI, encompassing both numerical methods and experiments, are provided in the recent work of Huang [20].

Herein, three numerical methods, namely SPH for wave modeling, the finite element method (FEM) for structural response, and latin hypercube sampling (LHS) for sampling structural parameters, are employed to facilitate forward uncertainty quantification (UQ). While SPH, FEM, LHS, and UQ are all well-established methods with extensive development in their respective fields, the novelty of this work lies in coupling these components to address uncertainties in the system structural response. Moreover, most of the works where SPH has been coupled with a structural solver have been limited to the adoption of monolithic rigid structures (cubes, cylinders etc.) or flexible beam structures. In this study, SPH is coupled offline with OpenSees [33]. The reason for employing offline coupling is that, while it is possible to use SPH for the entire WSI modeling process, the SPH structural model is computationally expensive and time-consuming compared to using the OpenSees model. Additionally, within the context of the SPH framework, UQ has seen limited integration previously. More specifically, this work explores UQ in the structural response under wave loading. While the framework has been presented to study the WSI in flume, the framework itself is generalizable. For example, when addressing variations in structural parameters, such as shape, size, or orientation. These variations are integral to the processes of structural design and optimization. By coupling SPH with OpenSees and integrating UQ analysis, the computational efficiency of the modeling process can be substantially enhanced, particularly when evaluating multiple structural configurations. Such uncertainties can have a critical impact in real-world decisions. The developed framework can be directly employed to explore these applications. This work further serves as a precursor to potential developments of a surrogate model-driven digital wave flume that could enable the incorporation of more extensive probabilistic wave conditions in the future.

The paper is structured as follows: Sect. 2 outlines the basic theoretical formulations related to SPH and UQ, followed by validation with experimental and OpenFOAM results from the literature in Sect. 3. Section 4 provides a discussion on the force calculation and probabilistic structural response. Finally, the work concludes with a discussion of the conclusions and outlines focus areas for the future.

## Numerical methods

This section provides a brief overview of the numerical methods, governing equations and UQ techniques employed to quantify uncertainties within the complex WSI system.

### Smoothed-particle hydrodynamics

This work adopts the weakly compressible Smoothed Particle Hydrodynamics (WCSPH) approach to model wave generation and propagation within the numerical flume. This choice is driven by the versatility offered by the scheme, which leverages a Lagrangian description of motion and eliminates meshing. This mesh-less Lagrangian approach provides significant flexibility when simulating nonlinear and fragmented flows, particularly in the presence of a free surface. This is especially advantageous for scenarios involving wave breaking, overtopping, and impact flows on structures. The WCSPH open-source DualSPHysics solver [13] with graphic processing unit (GPU) acceleration, is utilized in this work.

#### Governing equations

The continuous Lagrangian form of the mass and momentum conservation equations considered herein are,

1 $$\begin{aligned} \begin{aligned} {\frac{D\rho }{D t}}&=-\rho \nabla \cdot \textbf{u} \\ {\frac{D\textbf{u}}{D t}}&=-{\frac{1}{\rho }}\nabla P+\nu \varDelta \textbf{u} +\textbf{g} \end{aligned} \end{aligned}$$

where $\rho$ is the density, $\textbf{u}$ is the velocity vector, $\textbf{g}$ are the body forces, *P* is the pressure, and $\nu$ is the kinematic viscosity evolved in time *t*.

#### SPH discretization

In SPH, the continuous approximation (or integral representation) of any generic, sufficiently smooth spatial function $\phi \left( \textbf{r}\right)$ is expressed as the convolution of the function with a smoothing kernel,

2 $$\begin{aligned} \langle \phi (\textbf{r}) \rangle =\int _{\varOmega }\phi (\textbf{r}^{\prime })W(\textbf{r}-\textbf{r}^{\prime },h) \ \text {d}  \textbf{r}^{\prime } \end{aligned}$$

where $\textbf{r}$ is the position vector, $\textbf{r}^{\prime }$ is the position within the volume $\varOmega$ defined by *h* the characteristic smoothing length and $W(\textbf{r}-\textbf{r}^{\prime },h)$ is the smoothing kernel function. SPH uses the smoothing kernel function or simply kernel to calculate the weighted and averaged contributions of the particles within a cut-off distance of $\kappa h$ where $\kappa$ is a kernel specific constant and defines the extent of the kernel support domain [10]. This volume averaging approximation is represented by the angled brackets $\langle \cdot \rangle$.

In SPH, the domain is discretized into computational nodes, or particles, that are assigned fluid properties such as mass, density, velocity, etc. The integral representation of the field function of Eq. 2 is discretized by summing the weighted contributions of the nearest neighbor particles within the $\kappa h$ support radius. This implies that for any arbitrary particle *a*, the approximation reads,

3 $$\begin{aligned} \langle \phi (\textbf{r}_{a}) \rangle =\sum _{b=1}^{N_\text {p}}\frac{m_{b}}{\rho _{b}}\phi (\textbf{r}_{b})W_{a b} \end{aligned}$$

where *a* and *b* are the interpolating and neighboring particles, $N_\text {p}$ is the number of particles within the domain with $b = 1, \cdots , N_\text {p}$ neighbors, $m_{b}$ is the mass and $\rho _{b}$ is the density of the neighboring particle *b* that defines the volume of each particle as $V_{b}=\frac{m_{b}}{\rho _{b}}$. Herein, ${W}_{a b}\,\equiv \,{W} \left( \textbf{r}_{a}-\textbf{r}_{b},h \right)$ is the discrete kernel function. Note that the brackets $<.>$ that denote approximation will be dropped in the rest of the paper for brevity.

This work in particular, uses the Wendland $C^2$ kernel function implemented in DualSPHysics [52] that reads,

4 $$\begin{aligned} W(r,h)=\alpha _{d}\Biggl \{\left( 1-\frac{q}{2}\right) ^{4} \left( 1+2q\right) \;\;0\le \,q\,\le 2\, \end{aligned}$$

where

5 $$\begin{aligned} q={\frac{|{\textbf{r}}-{\textbf{r}}^{\prime }|}{h}} = {\frac{|r_{ab}|}{h}} \end{aligned}$$

and $\alpha _{d}$ is a normalization constant that depends on the spatial dimensions of the problem with $\kappa =2$.

Following the above discretization technique, the work uses the weakly compressible SPH formulation implemented in DualSPHysics [13] to obtain the following form of the governing equations,

6 $$\begin{aligned} \begin{aligned} \frac{D\rho _{a}}{D t}&=-\rho _{a}\sum _{b=1}^{N_\text {p}}\frac{m_{b}}{\rho _{b}}\textbf{u}_{a b}\cdot \nabla _{a}W_{a b} + \mathfrak {D}\\ {\frac{D{\textbf{u}}_{a}}{D t}}&=-\sum _{b=1}^{N_\text {p}}m_{b}\left( {\frac{P_{b}+P_{a}}{\rho _{a}\rho _{b}}}+\varPi _{a b}\right) \nabla _{a}W_{a b} + \textbf{g} \end{aligned} \end{aligned}$$

where $\mathfrak {D}$ is a density diffusion term added to dissipate checkerboarding, defined as a second order derivative of the hydrostatic density [16], and $\varPi _{a b}$ is an artificial viscosity [37] term given by,

7 $$\begin{aligned} \varPi _{a b}= {\left\{ \begin{array}{ll} -\frac{ \alpha \overline{c_{ab}}\mu _{ab} }{\overline{\rho _{ab}} } &  \text {if } x \ge 0 \\ 0 &  \text {if } x < 0 \end{array}\right. } \end{aligned}$$

and $\alpha$ is a parameter which can be tuned to provide sufficient numerical dissipation. In this work, following Altomare et al. [2], the value of $\alpha$ is set to 0.01 to provide sufficient dissipation for numerical stability in wave and wave loading flume applications.

A barotropic equation of state (EOS) [36] closes the system of Eq. 6 by relating pressure to density as

8 $$\begin{aligned} P = {\frac{c_{0}^{2}\rho _{0}}{\gamma }}\left[ \left( {\frac{\rho }{\rho _{0}}}\right) ^{\gamma }-1\right] \end{aligned}$$

where the polytropic index $\gamma = 7$, the initial fluid density is $\rho _{0} = 1000$ kg $\textrm{m}^{-3}$ and the initial speed of sound is defined as $c_{0} = c \left( \rho _{0} \right)$ within the incompressible limit of $Ma=0.1$ by imposing $c_{0} =10\sqrt{gH}$ where H is initial water height at rest.

The symplectic position Verlet scheme [23] is employed for time integration in this work. Due to the Lagrangian nature of SPH a second order accurate time integration scheme is required, thus the velocity step is updated at $n+1/2$ using the velocity Verlet half step, as given by:

9 $$\begin{aligned} \textbf{u}^{n+1/2} = \textbf{u}^{n} + \frac{1}{2} \varDelta t \ \textbf{a}^{n} \end{aligned}$$

The position is updated according to symplectic position Verlet scheme,

10 $$\begin{aligned} \textbf{r}^{n+1} = \textbf{r}^{n} + \varDelta t \ \textbf{u}^{n} + \frac{1}{2} \varDelta t^2 \ \textbf{a} \end{aligned}$$

with $\textbf{a}=\left( \frac{D\textbf{u}}{D t} \right) ^{n}$. Furthermore, the density evolution is calculated using the half time steps of the symplectic position Verlet scheme [39],

11 $$\begin{aligned} \rho ^{n+1/2} = \rho ^{n} + \frac{1}{2} \varDelta t \ \mathscr {D} \end{aligned}$$

where $\mathscr {D}=\left( \frac{D\rho }{D t} \right) ^{n}$ represents the rate of change of density at step *n*.

In this paper, the dynamic boundary condition (DBC) [8] is utilized to impose solid wall boundary. Boundary particles are subject to the continuity equation to evolve the density and thus impose a no-penetration condition.

Boundary particles are allowed to move according to externally prescribed motion such as a prescribed motion or wavemaker. The piston wavemaker generates waves by imposing the following specific motion function derived from the Rayleigh approximation,

12 $$\begin{aligned} \eta (x_s, t) = H \, \text {sech}^2 \left[ k \left( c \left( t - \frac{T_f}{2} \right) + 2 \sqrt{\frac{H(H+h)}{3}} - x_s \right) \right] \end{aligned}$$

where, $2\sqrt{H(H+h)/3}$ represents half of the wavemaker stroke based on the assumption that wavemaker motion starts from $x=0$; *k* represents the outskirt coefficient, *c* denotes the wave celerity which is a function of gravity (*g*), wave height (*H*), and water depth (*h*); $T_f$ indicates the time required to generate the solitary wave and $x_s$ the displacements of the wavemaker.

Over the years, several topical reviews [28, 30, 50, 53] and books [25–27] on the development and applications of SPH in coastal engineering have been written and reader is directed to the above for further information.

### Force exerted on the structure

An accurate estimation of forces is crucial for assessing the structural response within a wave flume. This work compares force calculations obtained from experimental results, SPH simulations, ASCE standards, and analytical expressions. The ASCE standards and semi-empirical employ wave velocities and heights to determine the time history of wave loading on the structure.

#### Force calculation in SPH

The post-processing tool within the DualSPHysics is employed to extract the forces exerted on the structures. The force tool allows for the extraction of horizontal forces acting on the column, as

13 $$\begin{aligned} F_{x,\text {SPH}} = \sum _{i} {P_i \ \text {d}x^2} \frac{\left( \textbf{x}_{ci} - \textbf{x}_i \right) }{ | \textbf{x}_{ci} | } \end{aligned}$$

where $\textbf{x}_{ci} = \left( x_c,y_x\right)$ is the column center coordinates, $P_i$ is the pressure and $\text {d}x^2$ represents summation over all infinitesimal areas. More detailed discussions, including relevant formulations on force-time history extraction and comparison of SPH with experimental results [18, 47] during impulsive provoked breaking waves can be found in Altomare et al. [2].

#### American Society of Civil Engineers (ASCE)

ASCE/SEI 7-16 [5] discuss a practical method for estimating the maximum wave breaking force (or shock pressure) on vertical walls of buildings in coastal areas. The ASCE relations, also considered in this work, for calculating the maximum combined dynamic and static wave pressures $P_{\textrm{max}}$, are

14 $$\begin{aligned} P_{\textrm{max}}= \left( C_\text {p} + 1.2 \right) \gamma _\text {w}d_\text {s} \end{aligned}$$

The net breaking wave force per unit length of the structure, also known as the wave impact force $\left( F_{\text {ASCE}}\right)$ is given to be

15 $$\begin{aligned} F_{\text {ASCE}} = \left( 1.1 C_\text {p} + 2.4 \right) \gamma _\text {w} d_\text {s}^2 \end{aligned}$$

where, $C_\text {p}$ is the pressure coefficient which varies from 1.6 to 3.5 according to the category of structure, $\gamma _\text {w}$ is the unit weight of water and $d_\text {s}$ is the still water depth at base of structure.

#### Semi-empirical calculation

The semi-empirical approach considered splits the overall wave-induced force into a hydrostatic and a dynamic component, i.e., $F_{\text {total}} = F_{\text {static}} + F_{\text {dynamic}}$. According to ASCE standards [4], the static force calculation considers water density ($\rho = 1000\,\text {kg/m}^3$) and gravitational acceleration ($g = 9.81 \,\text {m/s}^2$), and computed as

16 $$\begin{aligned} F_{\text {static}} =\frac{1}{2} w \left( h_{b}-0.75 \right) \left[ \rho \times g \left( h_{b}-0.75 \right) \right] \end{aligned}$$

where, $h_{b}$ represents the gauge value obtained from the near structure wave gauge above the initial free surface; *w* represents width of the structure. This static force component represents the force exerted on the vertical wall of the structure by a presumably standing water at equilibrium. In addition, the dynamic force caused by the water column is obtained by integrating the velocity data over the height range. Thus, the dynamic force $F_{\text {dynamic}}$ is dependent on the effective velocity $V_{\text {eff}}$ and is given to be

17 $$\begin{aligned} F_{\text {dynamic}} =\frac{1}{2} \rho C_\text {d} A V_{\text {eff}}^{2} \end{aligned}$$

where, $C_\text {d}$ represents the drag coefficient, and *A* indicates the surface area of the object normal to the flow.

### One-way coupling method

**Fig. 1:**
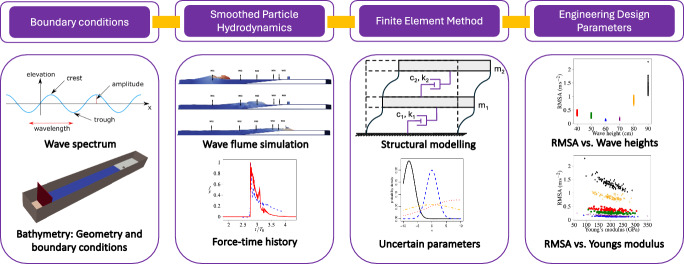
Illustration of the one-way coupling

The one-way coupling presented in this work is visually illustrated in Fig. 1. The work demonstrates the applicability for solitary waves but provides a general framework for other types of wave scenarios (regular, irregular, focused waves, etc.) with structures where the response of the structure does not significantly alter the hydrodynamics, i.e., with small displacements.

Herein, due to the very small deformations of the structure, we consider an offline coupling approach. The SPH solver propagates the wave and estimates forces that are obtained on the structure using the above-mentioned implementations in DualSPHysics. Thus, the temporal dynamics onto the structure by the wave (forces in our work) are recorded on an external data file. The FEM structural solver obtains these temporal forces exerted on the structure by the data file and imposes additional constrains. Note that, the geometrical characteristics of the structure in both solvers are identical and the zero displacement constrain at the base of the structure is only imposed in the FEM solver. As the response of the structure is two orders of magnitude smaller that the SPH discretization, there is no requirement to exchange the displacement with the SPH solver, resulting in a weak off line coupling. This approach has been applied in SPH successfully in Pringgana et al. [40]. The wave forces along with uncertainties in the material parameters are further used in the determination of structural response using FEM. The overall outputs of the numerical framework leads to engineering design parameters (EDP) such as root mean square acceleration (RMSA).

### Uncertainty quantification (UQ)

UQ is combined with structural dynamic analyses to estimate the probabilistic response of structures. This work considers the widely adopted latin hypercube sampling (LHS) as it facilitates an efficient exploration of the parameter space by systematically partitioning the parameter ranges into equi-probable intervals and selecting samples such that one sample is placed in each interval of each parameter dimension [32]. The resulting LHS samples ensure a more uniform and representative distribution of samples across all dimensions, effectively preventing sample clustering.

Without loss of generality, the *n* input parameters can be given to be $p_{i} \ \forall \ i=1,2,..,n$ and characterized by a specified range and divided into *q* equi-probable intervals or bins. The interval width can be given to be

18 $$\begin{aligned} \mathrm {Interval~width}={\frac{\textrm{Max}(p_{i})-\textrm{Min}(p_{i})}{q}} \end{aligned}$$

where, $\textrm{Max}(p_{i})$ and $\textrm{Min}(p_{i})$ are maximum and minimum value possible for the parameter $p_{i}$, respectively, *q* denotes the desired number of samples. The $q-$random permutations of the integers from 1 to *q* (inclusive), for each parameter $p_{i}$, are generated using a random permutation function, namely the Fisher-Yates shuffle algorithm [14].

The LHS matrix *L* is further created, which contains the LHS samples in domain of $[0,1]^n$. The dimension of *L* is $q \times n$ and each row corresponds to a sample point in the *n*-dimensional input space, with $L_{i}=(L_{i1},L_{i2},..,L_{i n})$, where $L_{i j}$ is the value of the *j*-th parameter in the *i*-th sample. The LHS sample matrix is constructed using the generated random permutations and the intervals. The elements of the LHS matrix *L* are mapped to the actual parameter values based on the intervals created using

19 $$\begin{aligned} p_{i j}=\textrm{Min}(p_{j})+(L_{i j}-1)\times \mathrm {Interval~width} \end{aligned}$$

where, $p_{i j}$ is the mapped value of the *j*-th parameter for the *i*-th sample.

Given the randomness in the individual structural properties (here depicted as a vector of properties) $\textbf{p}_\mathrm{{str}}$ and external forces $\textbf{F}$, the probability density function (PDF) of the structural responses of interest like root mean squared acceleration (RMSA), $\textbf{A}$, is computed as

20 $$\begin{aligned} \xi (\textbf{A}) = \int \xi (\textbf{A}| \textbf{p}_\mathrm{{str}},\textbf{F}) \ \xi (\textbf{p}_\mathrm{{str}})\xi (\textbf{F}) \ \textrm{d}\textbf{p}_\mathrm{{str}} \ \textrm{d}\textbf{F} \end{aligned}$$

where $\xi (\cdot )$ denotes the PDF and $\xi \left( \textbf{Y} | \textbf{X}\right)$ denote the conditional PDF of any $\textbf{Y}$ given $\textbf{X}$. Note here that $\textbf{p}$ is used to denote the set of all properties where uncertainties can exist. Some examples include Youngs’ modulus, Poisson ratio etc. Without additional randomness considered, the first term in the integral becomes a Dirac-delta function, $\xi (\textbf{A}|\textbf{p}_\mathrm{{str}},\textbf{F}) = \delta (h(\textbf{p}_\mathrm{{str}},\textbf{F})=\textbf{A})$, where $h(\textbf{p}_\mathrm{{str}},\textbf{F})$ represents the system equation, i.e., a combination of wave flume and structural dynamic simulation model. The PDF is approximated by LHS and kernel density estimation (KDE) as

21 $$\begin{aligned} \hat{\xi }(\textbf{A}) = \frac{1}{q} \sum ^m_{i=1} K_h(\textbf{A}-A_i) = \frac{1}{q} \sum ^m_{i=1} K_h(\textbf{A}-h(p_\mathrm{{str},i},F_{i})) \end{aligned}$$

where $[{p_\mathrm{{str},i},F_{i}}]$ represents *i*-th sample obtained from LHS and $A_i$ is corresponding structural analysis outcome. $K_h(\cdot )$ is a kernel function and this work uses, the commonly used, Gaussian basis function [11].

## Validation and convergence study

The SPH results from this work are validated by comparison with experiments conducted at the hybrid tsunami open flume (HyTOFU) in Ujigawa laboratory at the Kyoto University (Japan) and simulations performed using the OpenFOAM volume of fluids (VoF) solver [12, 42, 49]. The experimental and OpenFOAM results used for validation are reported in Moris et al. [38].

### Experimental setup

**Fig. 2:**
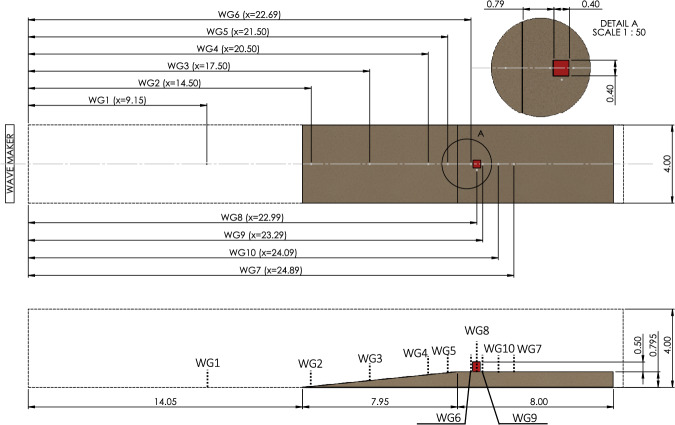
HyTOFU experimental set up including the location of wave gauges and the structure of interest. The dimensions are shown in SI units

The schematic and dimensions of the experimental setup are as shown in Fig. 2. The wave flume geometry considered was 32 m long, 4 m wide, and 4 m deep. The wave flume comprised of a flat bed spanning 14.05 m in length followed by a beach segment of constant slope ratio of 1:10 ($\theta$ = $5.71^\circ$), extending horizontally for another 7.95 m. Following the slope, the flume setup had an additional 8 m stretch of an even terrain situated at the height of 0.795 m. A single structure was situated on that flat terrain, 0.79 m beyond the end of the slope. The structure considered was of dimensions 0.4 m $\times$ 0.4 m $\times$ 0.5 m.

The free-surface elevations at ten different locations were measured using wave gauges (WG). The WG 1–6 were located upstream; WG 7, 9, and 10 were located downstream and in close proximity to the back of building; WG 8 was located adjacent to the building. The detailed information on the experimental setup, including information about the instrumentation and working, can be found in Moris et al. [38].

### SPH simulation setup

**Fig. 3:**
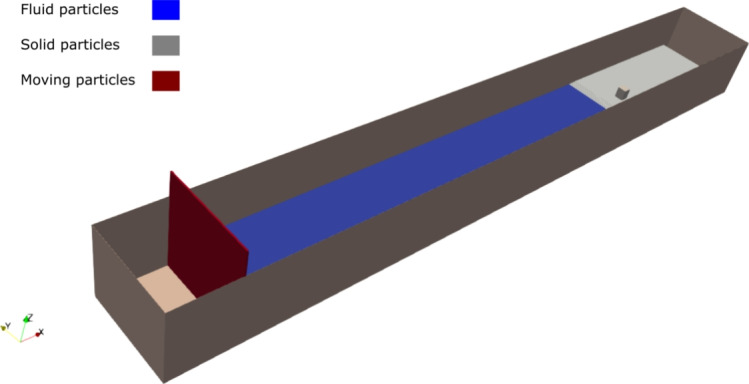
DualSPHysics setup of the HyTOFU wave flume. A depiction of the moving wall (red), solid walls (gray) and water (blue) Color figure online

A digital twin of the experimental flume has been reconstructed for numerical the simulation using DualSPHysics. The domain, shown in Fig. 3, is discretized into wall and fluid particles. The wall particles (gray) use the dynamic boundary conditions (DBC) [8] and represent the walls and the structure, while the red particles represent the moving particles associated with the piston wavemaker. The blue particles represent the water domain.

The initial water depth is 0.75 m and a solitary wave of 0.40 m is considered for validation. Further on, solitary waves of 0.40–0.90 m are considered for more detailed analysis. For the SPH simulations, the numerical speed of sound is automatically calculated by $c_{0}=10\sqrt{gH_{init}}$ where $H_{init}$ is the water depth at rest. The smoothing length is set to $h=2\textrm{dp}$ where $\textrm{dp}$ is the initial particle spacing. Herein, the density diffusion term of Fourtakas et al. [16] is employed to reduce pressure oscillations and an artificial viscosity of Monaghan [37] with $\alpha =0.01$ is used to ensure numerical stability. The initial water density is set to $\rho =1000$ kg $\textrm{m}^{-3}$. A detailed discussion on the method of solitary wave generation can be found in Sampath et al. [44].

The upstream of the flume consists of a moving wall and is discretized in SPH using the dynamic boundary conditions (DBC) [8] with a prescribed boundary velocity. The fixed sidewalls are represented by a set of fixed particles by solving the continuity equation to impose no penetration, and obtain a pressure from Eq. 8. This approach is computationally efficient, with the density and pressure computed simultaneously and thus resulting in substantial computational time savings. DBC has been used widely in solving coastal engineering problems by discretizing complex 3-D geometries without the need for complex mirroring techniques or semi-analytical wall boundary conditions [1, 54]. All the walls and building surfaces in this setup are treated with DBC.

### Numerical convergence

A convergence study is performed for the initial wave height of $\textrm{H}=0.4$ m. The convergence study is considered by varying the initial particle spacing $(\textrm{dp})$ used in the SPH simulations. Following the work of Altomare et al. [3], Roselli et al. [43], a minimum of four particles is deemed necessary to accurately represent wave height $(\textrm{H})$, and thus aiming for $\mathrm {H/dp} \ge 4$. The convergence study is considered for $\mathrm {H/dp} = 4, 16, 32$, and the resulting errors and simulation times are tabulated in Table 1.

**Table 1:** Numerical convergence

dp (m)	H/dp	Number of particles	Error in peak height (%)	Error in wave arrival time (%)	Simulation time
0.1	4	91,791	8.87	$-$2.20	78.51 s
0.025	16	4,000,681	1.96	$-$3.14	1.66 h
0.0125	32	29,818,849	1.86	1.57	30.7 h

**Fig. 4:**
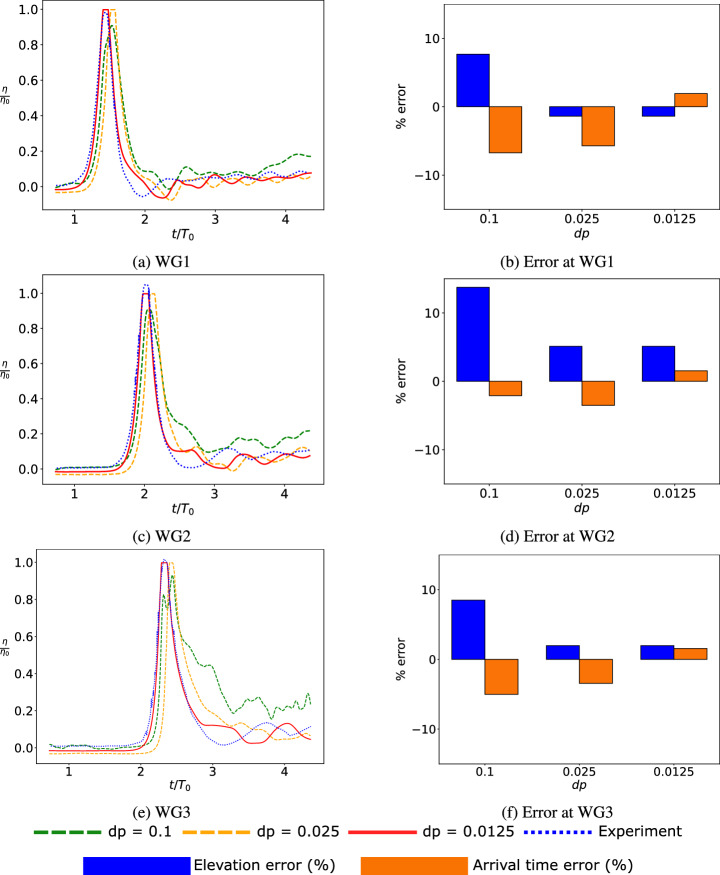
Mesh convergence study of SPH results for WG 1–3: normalized free-surface elevation obtained from SPH simulations are compared with experimental results from Moris et al. [38] for different inter-particle distances ($\textrm{dp}$) (left); resulting error, in comparison with experiments (right). $\eta$ and $\eta _0$ represent the wave height and characteristic wave height ($\eta _0$ = 0.4 m), respectively. *t* represents simulation time (12 s in total) and $T_0$ represents the characteristic time ($T_0 = 2.747$ s)

Initially, a value of $\textrm{dp} = 0.1$ m is chosen, resulting in four particles discretizing the wave height. This results in an error of 8.87% in the peak wave height and $-$2.2% in the wave arrival time. Subsequently, the inter-particle distance was reduced to $\textrm{dp} = 0.025$ m, resulting in a $\mathrm {H/dp} = 16$. This refinement was reflected in a reduced error of 1.96% in peak height. However, the wave arrival time continued to exhibit a deviation of $-$3.14% compared to the experimental data. Further reduction in the inter-particle distance to $\textrm{dp} = 0.0125$ m results in a $\mathrm {H/dp} = 32$. This level of refinement employing $\textrm{dp} = 0.0125$ m demonstrated superior agreement with the experimental data. It exhibited an error of 1.86% in peak height and 1.57% in wave arrival time. The total simulation time for this refined setup was 30.7 h. While there is not substantial change in the accuracy between $\textrm{dp} = 0.025$ m vs. $\textrm{dp} = 0.0125$ m, the latter results in at least 32 particles across the dimension of the structure and thus facilitates more accurate evaluation of the forces on the structure. The SPH results, i.e., free-surface elevation, for varying inter-particle distances, are compared with experimental results [38] in Fig. 4. As illustrated in Fig. 4, the dimensionless wave heights measured at WG1–3 are presented, where $\eta$ represents the wave height, and $\eta _0$ represents the characteristic wave height, which was selected as 0.40 m. *t* represents the time, and $T_0$ indicates the characteristic time, determined by the ratio of characteristic wavelength (*L*) to characteristic velocity (*C*), i.e., $T=L/C$. When the wave paddle generates 0.40 m wave height, the corresponding wavelength and velocity are selected as the characteristic wavelength and velocity and provided in Table 2. Figure 4 also shows the errors in free-surface elevation and wave arrival times compared against wave flume experiments [38]. As evident, the reduction in the inter-particle distance significantly improves the accuracy in the prediction of the wave arrival times.

**Table 2:** Wave paddles configuration

Wave height (m)	Wave length (m)	Celerity (m $\text {s}^{{-1}}$)	Wave height (m)	Wave length (m)	Celerity (m $\text {s}^{{-1}}$)
0.4	9.22634	3.35879	0.7	7.83151	3.77154
0.5	8.60361	3.50179	0.8	7.57411	3.89942
0.6	8.16210	3.63916	0.9	7.36769	4.02324

### Validation

The validation of the developed model has been conducted through comparison of the SPH results, discussed here, with experiments and the volume of fluid (VOF) simulations. The OpenFOAM (using VOF) simulations and experiments are not conducted as a part of this work but the results presented by Moris et al. [38] are used. For validation, the solitary wave of initial height of 0.40 m is considered.

**Fig. 5:**
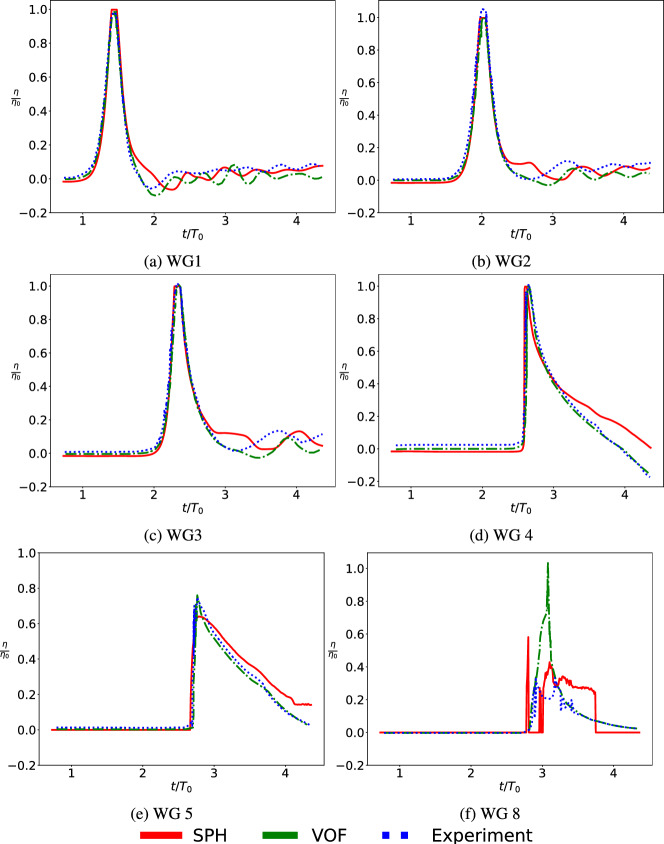
Validation of SPH simulation through comparison of normalized free-surface elevation at the wave gauges **a** WG 1, **b** WG 2, **c** WG 3 **d** WG 4 **e** WG 5 **f** WG 8. $\eta$ and $\eta _0$ represent the wave height and characteristic wave height ($\eta _0$ = 0.4 m). *t* represents simulation time (12 s in total) and $T_0$ represents the characteristic time ($T_0 = 2.747$ s). Here the comparison is between SPH (current work) with results from Moris et al. [38], namely VOF (OpenFOAM) and wave flume experiments. The results are compared only for the wage gauges upstream of the structure

As illustrated in Fig. 5, the normalized free surface elevation in the current work using SPH exhibits excellent agreement with the WG measurements in the initial flat and sloping section (WG 1–3) and satisfactory (WG 4–5) upstream of the building. The wave gauge positioned at the side of the building (WG8) exhibited better agreement with the experiments than VOF simulations. However, a limitation of the SPH approach used here, is that it does not provide variable resolution. A higher resolution can improve the wave gauge readings near to the structure.

**Fig. 6:**
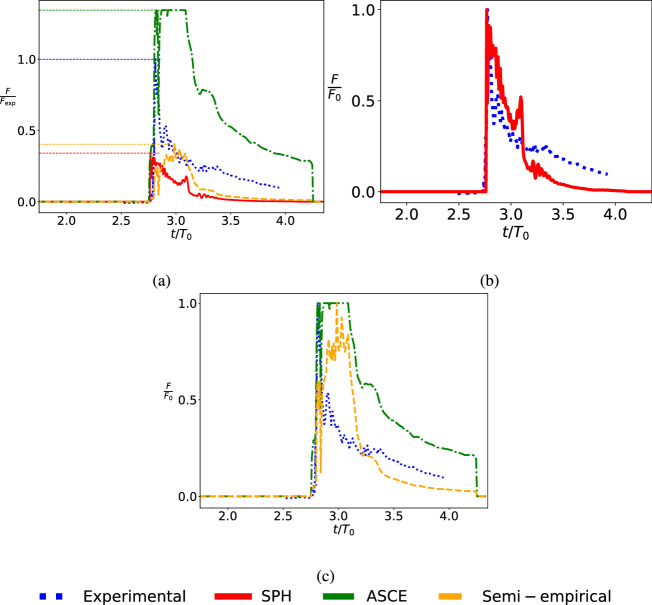
Comparison of non-dimensionalized force-time history acting on the structure. Comparison between **a** SPH (current work) with results from Moris et al. [38], namely wave flume experiments, ASCE standard and semi-empirical relations. **b** SPH (current work) with experiments results. **c** Experiments results, ASCE standard, and semi-empirical relations, *t* represents simulation time (12 s in total) and $T_0$ represents the characteristic time ($T_0 = 2.747$ s)

Figure 6 shows the comparison of the normalized forces. The comparison uses measured forces during experiments [48] with the force calculations outlined earlier from ASCE standards, semi-empirical calculations and the SPH simulations in the current work.

The force, represented as *F*, is non-dimensionalized using the peak force from different methods.

Force in Fig. 6a is normalized by the experimental peak force, represented by $F_{\textrm{exp}}$. In Fig. 6b and Fig. 6c, $F_0$ represents the peak force obtained from the results of different methods, with each method’s peak force used to normalize its own results. From Fig. 6a, it can be observed that the ASCE standards significantly overestimate the forces while both semi-empirical and SPH results underestimate the force-time history and the peak force acting on the structure. However, Fig. 6b shows that the SPH force decay trend matches the experimental force decay trend. Figure 6c indicates that neither the semi-empirical methods nor the ASCE standards can capture the decay trend. Besides, it is pertinent to note here that the validation is for a case without wave breaking effects.

The overall agreement in terms of wave arrival time and wave elevation with the majority of wave gauges is deemed as a basic validation for the numerical model. Deficiencies of significance are observed in the force calculation. The semi-empirical expressions appear to have lower error, the case considered does not account for wavebreaking effects. Alongside overestimating the peak forces, ASCE standard also provides erroneous rate of reduction from peak value. Considering the flat peak, this is nearly a quasi-static rather than a dynamic loading. Further, the load on the structure does not reduce to zero as rapidly as observed in experiments and other methods. Thus, despite ASCE providing the best probable estimate of the peak force, the overall time history needs to be considered with caution. SPH method, while largely underestimating the peak force, still captures the rate of change of force (or momentum) reasonably accurately.

Further, it is also pertinent to note that the forces are calculated at one point on the structure and could be further improved through calculation at multiple points and integration. This work is not aimed at improving the SPH method but at coupling with UQ to enable the quantification of uncertainties. The large variation also lends credence to the current work that aims to quantify the uncertainties. Considering the large variation between the forces calculated using the ASCE standard and those obtained from SPH simulations, quantifying these uncertainties is deemed more important than ever.

## Results and discussion

This section outlines the results and utilizes the validated SPH model. The section further outlines the discussions for varying wave heights, the resulting forces and the probabilistic structural response.

### Solitary wave propagation: wave heights

**Fig. 7:**
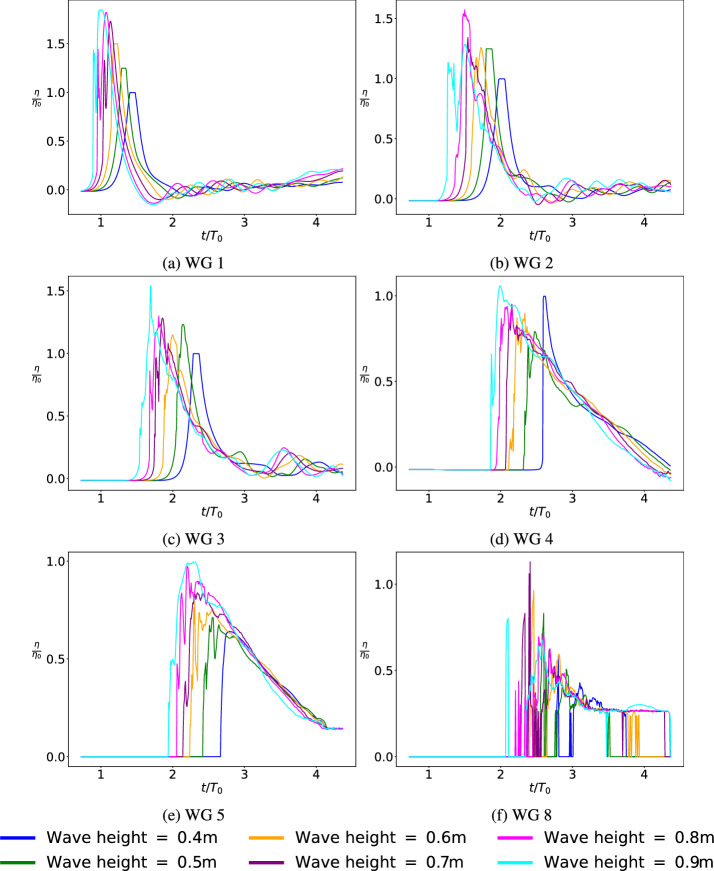
Normalized free-surface elevation observed at the wave gauge locations **a** WG 1 **b** WG 2 **c** WG 3 **d** WG 4 **e** WG 5 **f** WG 8 for varying initial wave heights, where $\eta$ represent the calculated wave height at the particular WG, $\eta _0$ represent the characteristic wave height ($\eta _0$ = 0.40 m), *t* indicates the simulation time, and $T_0$ represent a characteristic time ($T_0 = 2.747$ s)

The SPH setup discussed in the earlier section has been used for the simulation of solitary wave propagation in a wave flume. In this regard, considering same conditions outlined in the validation example, SPH simulations are setup with solitary waves of various initial wave heights, i.e 0.40–0.90 m at an interval of 0.10 m. Solitary waves at each height are listed in Table 2 with the corresponding wave length and celerity information. These solitary waves at different wave heights are generated based on Serre [45] wave theory by using the piston-type wavemaker. The normalized wave heights measured at the wave gauges 1–5 and 8 are shown in Fig. 7. It is pertinent to note that the solitary wave with initial wave height of 0.40 m has the least loss to wave height as it moves through the wave flume. The wave with initial wave height of 0.60 m or more nearly lose about 50% of wave height by the time the wave reaches the location of WG 5.

Appreciable wave heights are not measured at the wave gauges 7, 9 and 10 and thus not discussed here. However, only the waves with initial wave height of 0.80 and 0.90 m result in substantial overtopping.

### Forces on structures

**Fig. 8:**
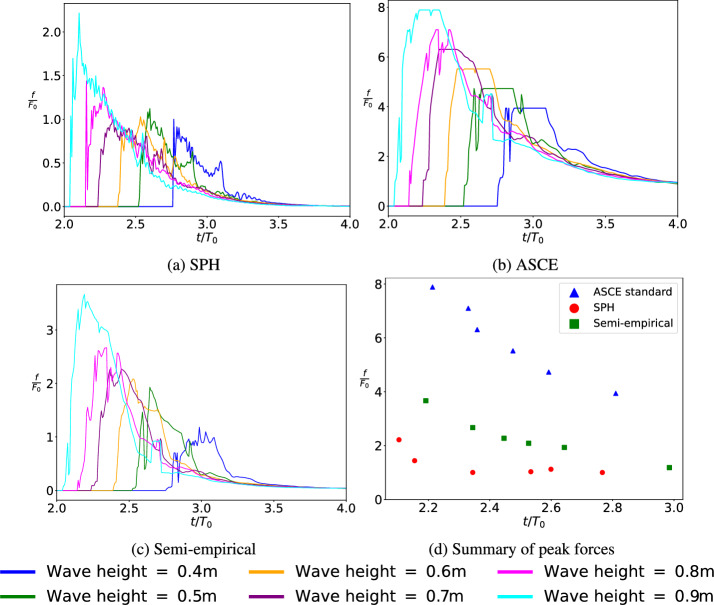
Normalized force-time history obtained from **a** SPH **b** ASCE standards **c** semi-empirical expressions **d** summary of peak forces. The summary extracts only the peak values from the SPH, ASCE and semi-empirical results. *f* represents the force measured/calculated, $F_0$ represent the characteristic force ($F_0 = 695$ N), *t* indicates the simulation time, and $T_0$ represent the characteristic time ($T_0 = 2.747$ s)

The forces are obtained through post-processing from SPH simulations and compared with the ASCE regulations and analytical expressions. The non-dimensionalized drag force-time histories for varying initial wave heights from 0.40 to 0.90 m are shown in Fig. 8. The peak force exerted on the structure where the wave height is 0.40 m, is chosen as the characteristic force ($F_0$ = 695 N). It is pertinent to note here that the ASCE standard and semi-empirical calculations consider the wave heights and velocities at the front of the structure. However, in contrast, SPH calculations consider all the faces of the structure in the calculation of forces and thus better represents the averaged overall force on the building [13]. However, in the current form, used in this work, SPH uses an offset distance to calculate these forces causing slight, but acceptable, inaccuracy in the peak forces.

As seen in Fig. 8, the forces predicted by ASCE standards are on the higher end, while SPH is on the lower end. This has also been discussed in the earlier section on validation in the discussion related to Fig. 6. The forces predicted using ASCE standards are about four times that predicted by SPH. This can be attributed to the overall area on which the forces act. While semi-empirical expressions demonstrate a better match with experiments, the same might not be translatable to all scenarios, in particular to breaking waves. Thus, semi-empirical solutions have limited applicability and needs to be considered carefully.

Further, the resulting normalized force-time plot, depicted in Fig. 7, reveals a behavior in contrast to the general assumption of direct proportionality between wave height and maximum drag force. The SPH simulations indicate that the wave loading is not strictly proportional to wave height, as seen through ASCE or semi-empirical calculations. While the wave arrival times are as expected across the board, with the 0.40 m wave arriving much later than a 0.90 m solitary wave, the maximum force does not increase proportionally as wave height changes from 0.40 to 0.90 m. As the wave height increases from 0.40 to 0.50 m, the peak forces increase; further it decreases for wave heights 0.60 and 0.70 m; to drastically increase again for wave heights 0.80 and 0.90 m.

From Fig. 7, it was observed that the solitary wave of 0.40 m continues to preserve most of the wave shape as it approaches WG 5; however, the wave heights still demonstrate a linear variation at WG 5. This is further substantiated in Fig. 14 in Appendix A where the wave of height 0.40 m can be characterized as a surging or collapsing wave. Similarly, Fig. 16 in Appendix A shows the wave as a spilling breaker and Fig. 17 in Appendix A as potentially a plunging breaker. The waves with wave heights of 0.80 m and 0.90 m also show significant overtopping effects. Further, Figs. 14, 15, 16 and 17 in Appendix A also demonstrate that the wave with height 0.40 m is a non-breaking wave; 0.70 m has a spilling effect; 0.90 m breaks as early as WG1-WG2 but transports a large volume of water. The varied behavior can, thus, be attributed to the nonlinear response of observed force vs. wave heights.

### Uncertainty quantification

Probabilistic structural analysis is performed to identify the range of structural responses considering the uncertainties in both structural properties and the wave heights. While the probabilistic analysis is computationally expensive compared to deterministic simulations, it provides more reliable estimates for practical engineering applications [17, 22, 31, 35].

Uncertainties in structural responses typically arise from two primary sources: the parameters associated with the structural behavior and those related to the wave mechanics. The former includes basic attributes such as mass, stiffness, damping of the structure and more intricate characteristics such as the modulus of elasticity and yield strength of the building’s individual components. The latter can be attributed as a variation in the wave forces, i.e wave heights, itself. This work leverages the developed NHERI SimCenter UQ engine [11, 34].

**Fig. 9:**
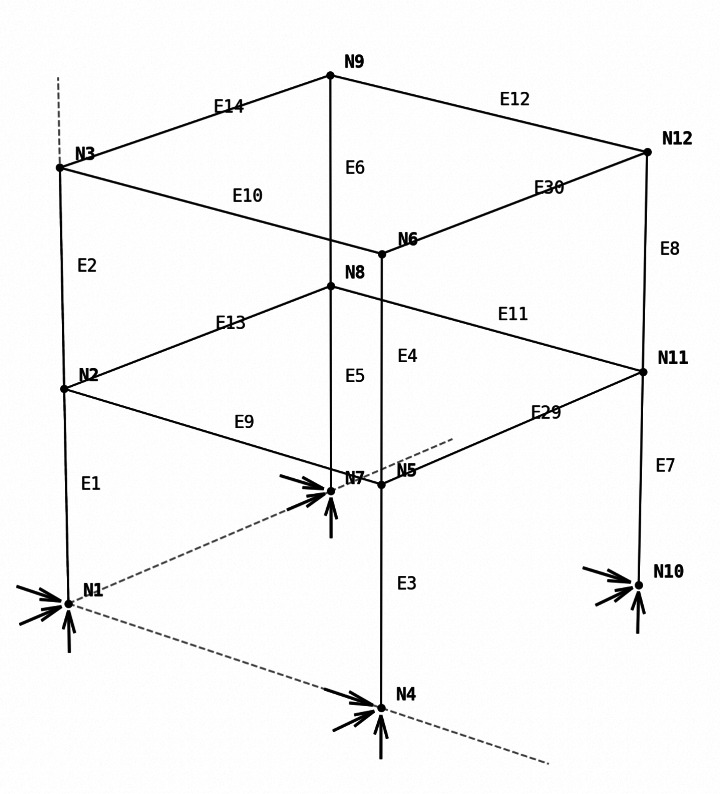
OpenSees beam-column model, constructed by W-section steel, and used in the structural analysis

**Table 3:** List of random variables for two-storey structure

Variable	Distribution type	Mean	Standard deviation
Yield strength (MPa)	Normal	413.685	82
W-section weight per length of column (N $\textrm{m}^{-1}$)	Normal	173.4	34
W-section weight per length of beam (N $\textrm{m}^{-1}$)	Normal	133.554	26
W-section weight per length of girder (N $\textrm{m}^{-1}$)	Normal	133.554	26
Young’s modulus for steel (GPa)	Normal	200	40

An OpenSees beam-column model, as shown in Fig. 9, is used to represent a two-storey structure consisting of column, beam, and girder sections, constructed using steel W-sections. The loading on the structure is derived from the wave loading history applied at each centroid node (i.e., storey level). This force history is obtained from earlier discussed methods: ASCE standard, semi-empirical expressions and SPH.

#### Sample generation

Considering the inputs as a random variable, a forward UQ method, namely LHS [11], is used for sample generation and aid in quantifying the propagation of the uncertainties. The engineering demand parameters (EDP) define the quantities of interest. In this work, peak floor displacement and root mean square acceleration (RMSA) are considered while additional user-defined EDP’s can also be added.

The LHS method is utilized to generate samples. A hundred sample points per parameter are required to analyze how structural variables change affect structural responses. Note, the number of samples was selected by performing a sensitivity study ranging from 10 to 200 samples per parameter to cover the entire variable distribution space and effectively capture the uncertainty characteristics of the structure, while maintaining computational cost to an affordable level. The structural random variables used in this work are specified in Table 3. A 20% coefficient of variation is considered to evaluate the probabilistic structural response. Further, uniform distribution is assumed for the wave parameters, i.e., wave heights over the discretized domain of 0.40–0.90 m, at intervals of 0.10 m.

**Fig. 10:**
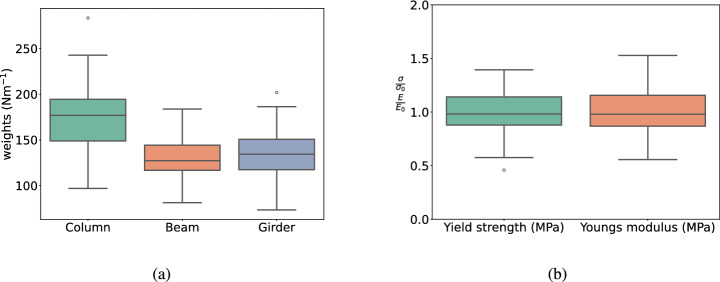
Distribution of structural properties considered: **a** column weight, beam weight, girder weight; **b** normalized yield strength ($\sigma _0 = 413.685$ MPa), and normalized Young’s modulus ($E_0 = 200$ GPa)

The boxplots in Fig. 10 depict the distribution of sample values generated using LHS method. It compares the distributions of column weight, beam weight, and girder weight and can be observed that the median of column weight is the largest, while the differences between the medians of beam weight and girder weight are relatively small. This suggests that columns may contribute significantly within the structure.

**Fig. 11:**
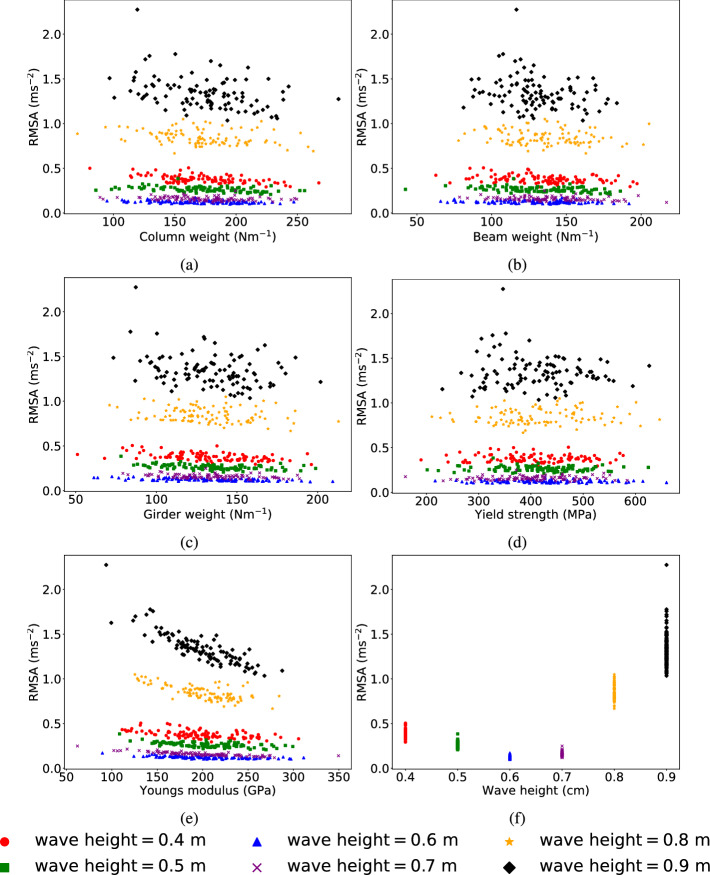
Two-storey structure’s root mean square accelerations (RMSA) in *x*-direction obtained using OpenSees plotted vs **a** W-section weight per length for columns (column weight), **b** W-section weight per length for beams (beam weight), **c** W-section weight per length for girder (girder weight), **d** Yield strength, **e** Young’s modulus, **f** Wave height

The relationships between the peak displacements, root mean square accelerations (RMSA) and the random variables, outlined in Table 3, are illustrated in Figs. 11 and 19 in Appendix A, respectively.

#### Peak floor displacement

Figure 19e in Appendix A shows a nonlinear and strong negative correlation between Youngs’ modulus and peak displacement in *x*-direction; a strong nonlinear trend in relation to initial wave heights. A general trend demonstrates little correlation between peak displacement and other parameters.

However, as shown earlier, the increase in wave height does not correspond to a proportional increase in the forces. Thus, the multi-dimensionality of the problem renders it hard to ascertain exact correlations across the spectrum by just using the peak displacement.

#### Root mean square acceleration (RMSA)

Considering the correlations shown between RMSA and the random variables in Fig. 11, the trend discussed for the Youngs’ modulus and wave height with respect to RMSA is consistent with the observations with peak displacement.

However, unlike as observed with peak displacements, the variations in RMSA versus random variables show a clear distinct behavior for each of the different wave heights considered. This further establishes that the initial wave heights significantly influence RMSA. The column, beam and girder stiffness, yield strength all show a weak nonlinear relationship with the RMSA. As expected, the overall RMSA decreases as Youngs’ modulus value increases.

In reality, the loads exerted on a structure are influenced by different aspects, including the dynamics of the environment, the configuration of the structure, and the material composition. This variability introduces complexity and uncertainty into the load-bearing dynamics of the structure. The RMSA analysis shown in Fig. 11 is based on the assumption of constant loads, whereas other distribution types reflect different structural responses that may occur. For example, static loads or uniformly distributed loads show characteristics of a normal distribution or uniform distribution, especially when the loads are symmetrically distributed. However, when considering irregularities in material properties or variations in foundation depths, modeling with other distributions, for example like beta distribution, is promising.

Furthermore realistic structural models should be explored to enable more accurate structural modeling and benchmarking. It is possible that the relationship between these structural parameters and RMSA can be strongly nonlinear for taller structures and warrants further investigation.

**Fig. 12:**
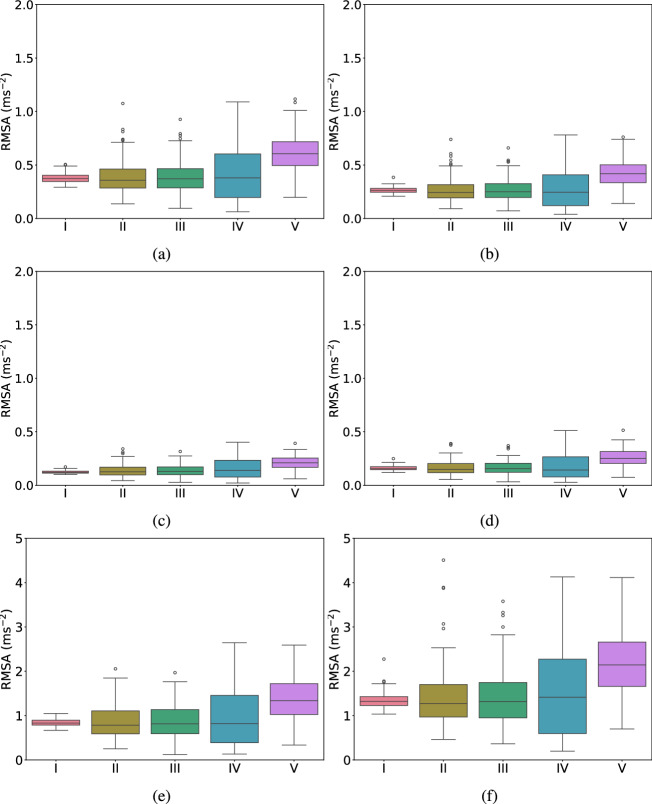
RMSA with different distribution including: (I) constant, (II) lognormal, (III) normal, (IV) uniform, (V) beta under varying wave heights: **a** 0.4 m, **b** 0.5 m, **c** 0.6 m, **d** 0.7 m, **e** 0.8 m, **f** 0.9 m

#### Wave loading uncertainty and its influence of probability distribution

In Sections 4.3.2 and 4.3.3, the correlation between structural variables and structural responses has been analyzed. In this section, the impact of uncertainty in the wave loading applied to the structure is further considered. Considering various input distributions for wave loading is important as in real-life, wave conditions are influenced by many factors, including wind speed, seabed topography, and meteorological conditions. These factors result in diverse statistical distributions for wave loads rather than a single deterministic value. Thus, the exact type of distribution required to describe the wave load remains uncertain and context-specific, highlighting a need for further research. Therefore, incorporating different distributions, compared to a single distribution, into the analysis provides a broader insight into uncertainties in wave loading on how those affect the accuracy and reliability of the results. Structural variables in this study are randomly selected and assumed to follow a normal distribution, consistent with Sect. 4.3.1. This study further analyzes the sensitivity of the choice of probability distribution for wave loading.

**Fig. 13:**
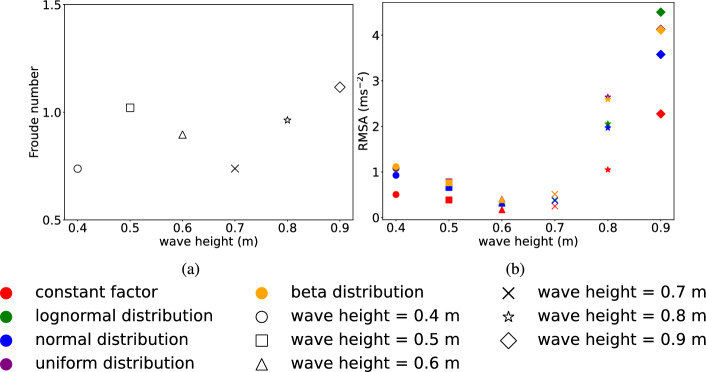
**a** Maximum Froude number under varying wave heights. **b** RMSA with different distribution including: constant, lognormal, normal, uniform, beta under varying wave heights: 0.40–0.5 m at intervals of 0.10 m

In this regard, lognormal (mean=1, standard deviation=0.2), normal (mean=1, standard deviation=0.2), uniform (min=0.4, max=1.6), and beta (alpha=5, beta=2, min=0.4, max=1.6) distributions are considered. The probability distribution parameters are considered such that the load values generated fall within the same overall range. This setup allows the distribution shape and characteristics to be the primary variables, enabling a clearer observation of the influence of distribution shapes and characteristics on structural responses. The diverse impacts of different distributions on structural responses are illustrated in Fig. 12.

The boxplot shown in Fig. 12 helps statistically visualize the variation of RMSA under different distributions and initial wave heights. It could be observed that the median lines in each distribution’s boxplot are quite close, except for the slightly higher median line in the beta distribution. This indicates that the system exhibits strong robustness to moderate random loads (inferred from initial wave heights) under different distributions, as the RMSA response to moderate shifts in magnitude remains consistent.

Further, the interquartile range (IQR) is used to represent the middle $50\%$ of the RMSA data’s distribution range, where the range between the upper quartile and lower quartile is the IQR. The upper whisker and lower whisker, respectively, represent the major distribution within the range of maximum and minimum values.

Under conditions of different wave heights with a uniform distribution applied to the loads in each case, the length from the upper whisker to the upper quartile is significantly longer compared to the length from the lower whisker to the lower quartile for each wave height. This phenomenon may be attributed to the asymmetry in structural response when the uniformly distributed load is applied as a random variable. Such asymmetry becomes particularly evident under higher load conditions, resulting in increased fluctuations in RMSA responses. Moreover, the application of uniformly distributed forces could induce extreme values in structural responses, causing the RMSA to predominantly cluster near the upper whisker.

Further, the circles in Fig. 12 mostly appear in when normal and lognormal distributions are considered. These circles represent outliers, indicating extreme RMSA values that exceed normal operational conditions. These extreme RMSA values need to be analyzed to determine whether the structure is critically stressed to the point of damage and/or failure.

Thus, analyzing the maximum values of RMSA produced by considering different wave heights and distributions could help to assess the maximum impact force that the building may experience under various scenarios and shown in Fig. 13b. The constant factor results in the smallest maximum RMSA values among all distribution conditions.

When the wave height is 0.80 m, the maximum value of RMSA generated by beta distribution applying to the wave load is 2.59211 $\textrm{ms}^{-2}$ which is almost 2.5 times of the RMSA generated by the constant factor.

When the wave height is 0.90 m, the maximum value of RMSA caused by lognormal distribution on wave load is 4.50665 $\textrm{ms}^{-2}$, which is also the maximum value of RMSA under six different wave heights with different distribution conditions.

Overall, when the wave heights are 0.40 m, 0.50 m, 0.60 m, 0.70 m, and 0.80 m, the maximum RMSA values generated by wave loads on the structure under Beta distribution and uniform distribution show very little discrepancy. These values are higher compared to the maximum RMSA values under normal distribution, lognormal distribution, and constant factor applied to the wave loads.

#### Correlation with Froude number

The Froude number is introduced in the study, which could indicate the correlation between the flow inertia force and the external force. Additionally, different Froude numbers indicate varying wave characteristics, which affect the magnitude and application of wave loads, thereby influencing the RMSA values of the structure.

The $V_\text {eff}$ from Eq. 17 was used in calculation of Froude number. The $V_\text {eff}$, here is defined as the average speed within the height range along the structure’s center line for each time step. Then, the maximum $V_\text {eff}$ value of different wave height are selected to calculate the Froude number, maximum Froude numbers for wave height ranging from 0.40 to 0.90 m, in intervals of 0.10 m, are plotted in Fig. 13a.

In Fig. 13a, when wave heights are 0.50 m and 0.90 m, the maximum Froude number exceeds 1, indicating supercritical flow conditions for these cases. In all other cases, the maximum Froude number is less than 1, indicating sub-critical flow. It is noteworthy that at the wave height of 0.90 m, where the maximum Froude number is approximately 1.116, the highest among all conditions depicted in Fig. 13a. Therefore, the Froude number indicates that wave speeds are highest in this case, resulting in the maximum wave loads and RMSA values.

Additionally, it is evident that the variation trend of the Froude number with wave height aligns consistently with the trend of force variation with wave height in Fig. 8a, and corresponds to the trend of RMSA variation with wave height in Fig. 13b, except when the wave height is 0.40 m. This discrepancy may be attributed to the lower wave height, resulting in a more stable flow regime.

## Conclusions and future work

The work provides a comprehensive numerical framework to estimate probabilistic structural response under wave loading conditions in a wave flume. The HyTOFU flume configuration is used in this regard as the standard wave flume configuration. However, the developed method is general and applicable to other geometries as well. As shown, even for a single wave flume geometry, structural variables significantly influence the structural response under different wave conditions. Deterministic analyses have a limited ability to consider multiple variables simultaneously, which makes it difficult to analyze combined effects that can easily lead to structural failure. Therefore, uncertainty quantification remains essential to extend the interpretation of wave flume results to real-world conditions, even though conducting uncertainty quantification analysis increases computational cost.

Under different wave conditions, the proposed framework integrates various structural parameters to calculate the probability distribution and variation trends of RMSA and peak displacement. Unlike deterministic analysis, which produces a single numerical result, this framework quantifies the structural risk under variable conditions. By applying UQ to assess the sensitivity of different structural parameters, this framework identifies those with the most significant impact on the structural response. Thus, this framework could supports the optimization of structural parameters to meet design standards and improve resilience to extreme conditions. For example, coastal structures exposed to tsunamis could be analyzed using this framework. It allows the quantification of structural responses under varying wave conditions and assesses the combined effects of parameter variations on structural failure. This approach helps evaluate the reliability of structures under different material conditions during the design phase and provides guidance for improving stability and resistance to extreme events.

**Fig. 14:**
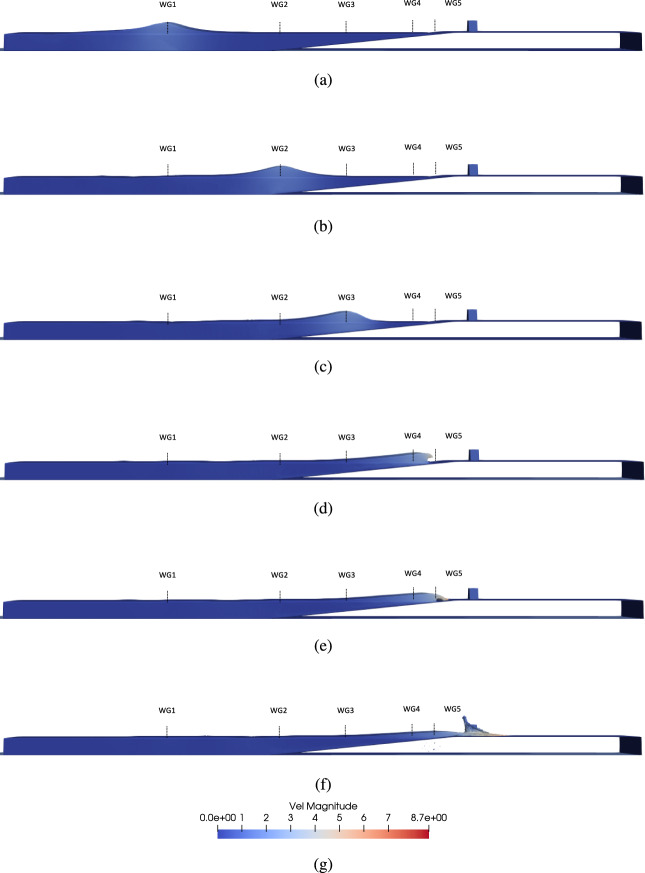
Propagation of the solitary wave for the initial wave height of 0.40 m

**Fig. 15:**
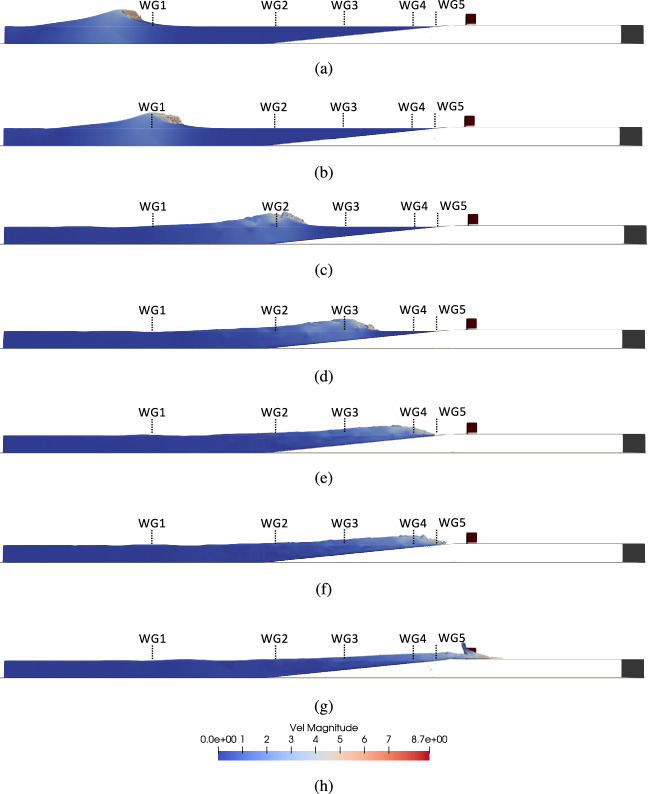
Propagation of the solitary wave for the initial wave height of 0.60 m

**Fig. 16:**
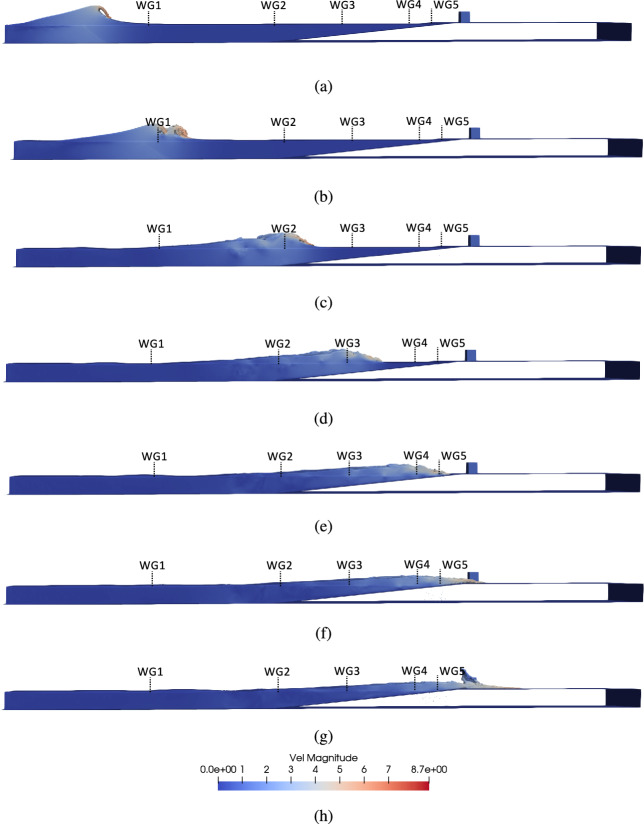
Propagation of the solitary wave for the initial wave height of 0.70 m

**Fig. 17:**
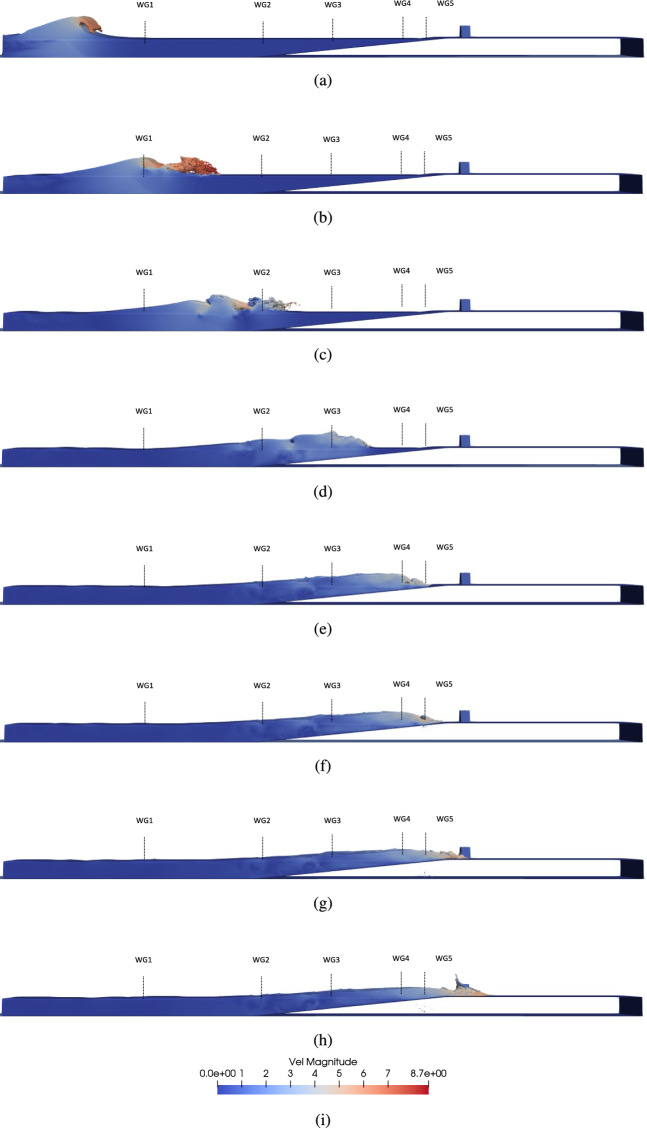
Propagation of the solitary wave for the initial wave height of 0.90 m

**Fig. 19:**
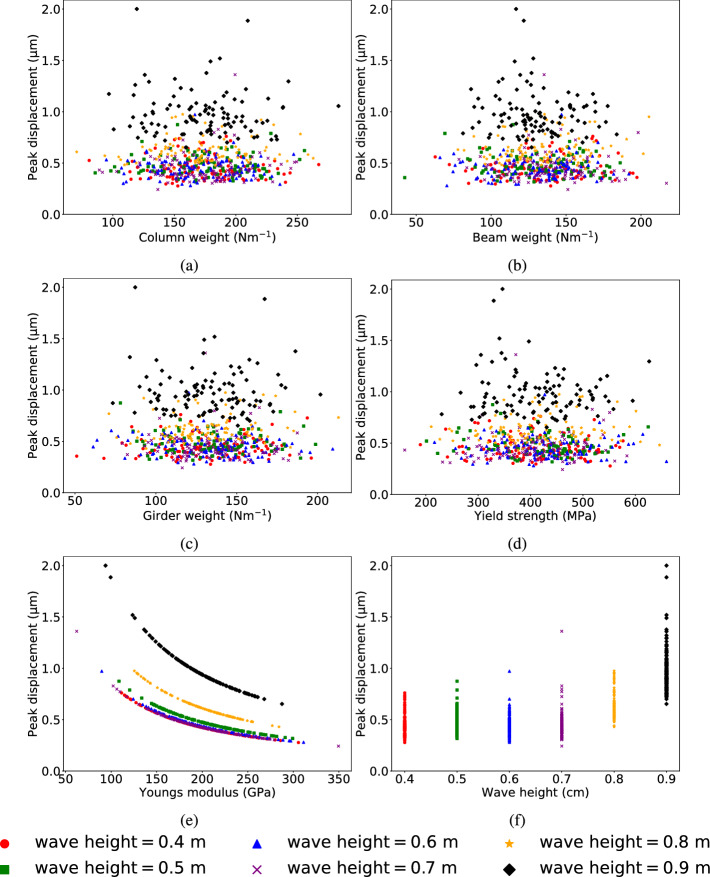
Two-storey structure’s peak displacements obtained using OpenSees vs. **a** W-section weight per length for columns (Column weight), **b** W-section weight per length for beams (Beam weight), **c** W-section weight per length for girder (Girder weight), **d** Yield strength, **e** Youngs’ modulus, **f** Wave height

While SPH for wave loading on a single structure nor FEM themselves are not novel, the novelty of this work lies in the coupling of different numerical methods, albeit one-way. The work demonstrates a nonlinear dependence on the initial wave height vs. the root mean square acceleration, as shown in Fig. 11f. This is further substantiated by the wave contours shown in Appendix in Figs. 14, 15, 16 and 17 where the wave breaking effects for medium-sized waves, of height comparable to still water depth, are shown.

Overall, the work provides a systematic method for uncertainty quantification and data curation for wave–structure interaction problems. The envisioned next steps of this work is in the usage of the developed models for surrogate modeling toward a true digital wave flume. Despite the development of digital wave flumes using CFD models, full 3-D high-fidelity CFD simulations remain computationally expensive. Consequently, digital wave flumes have remained simulation constructs rather than true digital twins. This necessitates the creation of surrogate models capable of providing a sufficiently fast and accurate representation of the system. Furthermore, a range of uncertainties significantly influence WSI problems, including initial wave conditions, air–water interaction, temperature, bathymetric surface roughness, and structural properties, among others. The computational cost of simulation techniques hinders extensive quantification of these uncertainties within the system. Conversely, the existence of a surrogate model could enable rapid evaluation of potential WSI scenarios. Considering the expense and time associated with experimental setup, such a surrogate model would not only facilitate the incorporation of uncertainties but also lead to more cost-effective and time-efficient experiment design. Further, considering extensive experimental data available with large wave flumes like those in Kyoto (Japan), Hannover (Germany), Oregon (USA) etc., this provides an excellent opportunity to develop digital twins of use to the community.

## Figures and Tables

**Fig. 18:**
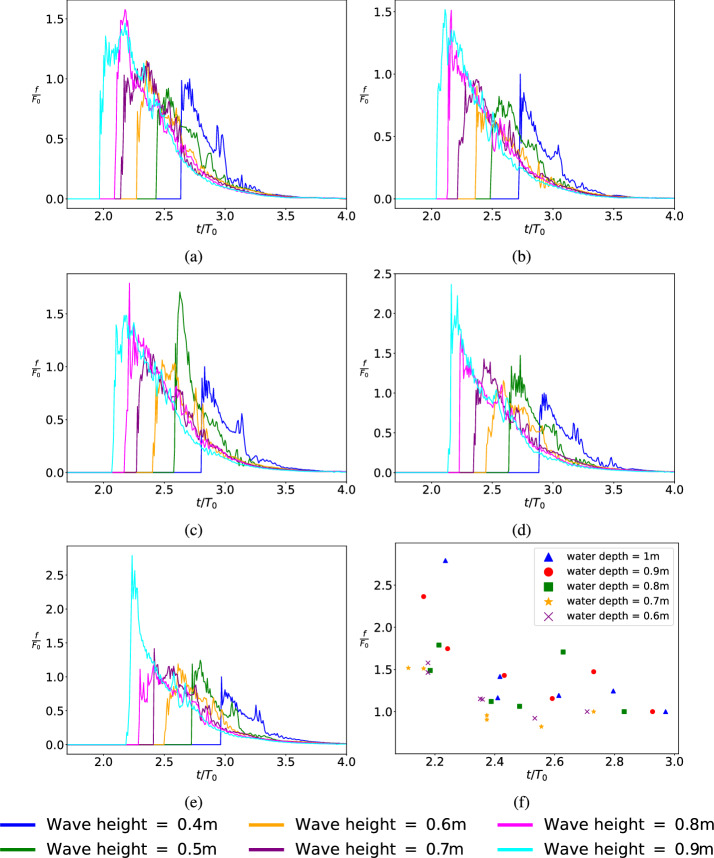
Normalized force-time history obtained from **a** water depth is 0.6 m **b** water depth is 0.7 m **c** water depth is 0.8 m **d** water depth is 0.9 m **d** water depth is 1 m **e** the summary extracts only the peak values from (**a**) to (**d**) results. *f* represents the force measured/calculated, $F_0$ represents the characteristic force ($F_0 = 695$ N), *t* indicates the simulation time, and $T_0$ represents the characteristic time ($T_0 = 2.747$ s)

## Data Availability

The input files, resulting data and plotting routines with documentation (presented in this work) have been shared through Zenodo (http://doi.org/10.5281/zenodo.14655913).

## Appendix A: wave height contours

The wave contours (side-view) are shown for three wave heights, namely 0.40 m (Fig. 14), 0.60 m (Fig. 15), 0.70 m (Fig. 16) and 0.90 m (Fig. 17). As discussed in the paper, the overall drag force on the structure reduces from 0.40 to 0.70 m and further increases to 0.90 m.

See Figs. 18 and 19.
